# The Relationship between Frailty Syndrome and Concerns about an Implantable Cardioverter Defibrillator

**DOI:** 10.3390/ijerph17061954

**Published:** 2020-03-17

**Authors:** Agnieszka Mlynarska, Rafal Mlynarski, Izabella Uchmanowicz, Czeslaw Marcisz, Krzysztof S. Golba

**Affiliations:** 1 Department of Gerontology and Geriatric Nursing, School of Health Sciences, Medical University of Silesia, Katowice 40-635, Poland; cmarcisz@sum.edu.pl; 2 Department of Electrocardiology, Upper Silesian Heart Centre, Katowice 40-635, Poland; rmlynarski@sum.edu.pl (R.M.); ralphie@o2.pl (K.S.G.); 3 Department of Electrocardiology and Heart Failure, School of Health Sciences, Medical University of Silesia, Katowice 40-635, Poland; 4 Department of Clinical Nursing, Faculty of Health Sciences, Wroclaw Medical University, Wroclaw 51-618, Poland; izabella.uchmanowicz@umed.wroc.pl

**Keywords:** implantable cardioverter defibrillator, concerns, frailty syndrome, anxiety, depression

## Abstract

Frailty syndrome may cause cognitive decline and increased sensitivity to stressors. This can result in an increased incidence of anxiety and depression, and thus, concerns about life with an implantable cardioverter defibrillator (ICD). The aim of the study was to assess the impact of frailty syndrome on the increase in the number of device-related concerns after the implantation of an ICD. Material and methods: The study sample was a group of 103 consecutive patients (85 M; aged 71.6 ± 8.2) with an implanted ICD. The ICD Concerns Questionnaire (ICDC) was used to analyze their concerns about life with an ICD, and the Tilburg Frailty Indicator scale (TFI) was used to diagnose frailty. Results: In the group of patients with an ICD implanted, 73% had recognized frailty (83.3% women, 74.1% men); the average point value was 6.55 ± 2.67. The total ICDC questionnaire score for the patients with an implanted cardioverter defibrillator was 34.06 ± 18.15. Patients with frailty syndrome had statistically (*p* = 0.039) higher scores (36.14 ± 17.08) compared to robust patients (27.56 ± 20.13). In the logistic regression analysis, the presence of frailty was strongly associated with the total questionnaire score (OR = 1.0265, *p* = 0.00426), the severity of the concerns (OR = 1.0417, *p* = 0.00451), and device-specific concerns (OR = 1.0982, *p* = 0.00424). Conclusion: Frailty syndrome occurs in about 80% of patients after ICD implantation. The presence of frailty syndrome was strongly associated with concerns about an implantable cardioverter defibrillator.

## 1. Introduction

Although treatment using an implantation cardioverter defibrillator (ICD) extends the lives of patients that are at a high risk of sudden cardiac death, it may also affect a patient’s quality of life and performance. Some patients may develop anxiety and depression, and these disorders are the most severe in patients who have experienced unnecessary and frequent device firings [1,2,3]. Unnecessary ICD interventions occur in 19%–35% of patients, and in most patients, the firing of an ICD is associated with very unpleasant sensations. According to the literature, ICD firing can have a negative impact on a patient’s functioning in the psychological sphere. Most researchers believe that the short-term accumulation of discharges and so-called electrical storms (at least three firings within 24 h) can lead to severe anxiety–depression disorders, which in extreme cases resemble post-traumatic stress disorder. The association of a neutral situation with an ICD firing strengthens the behaviors that are aimed at avoiding the factors that led to the device shocks. As a result, this leads to a significant limitation of various types of patient activity, for example, avoiding any physical effort due to the fear of firing or avoiding the places, people, and situations that are reminiscent of the circumstances in which the firing once occurred [4,5].

Frailty syndrome (FS) is a common clinical syndrome in the elderly that carries an increased risk of poor health outcomes, including falls, disability, hospitalization incidents, and mortality. Frailty syndrome is caused by subclinical disorders in the multi-organ systems that lead to the loss of homeostatic reserve and immunity. One of the central handicaps is the age-related loss of muscle mass and strength, sarcopenia, which is defined by a number of phenotypic criteria: slow walking, weak muscle strength, unintentional weight loss, low physical activity, and exhaustion. By definition, frailty syndrome also causes cognitive decline and increased sensitivity to stressors. This can result in an increased incidence of anxiety and depression, and thus, concerns about the new treatment method that is being used. Studies have shown that frailty syndrome is a major risk factor in cases of death and readmission in patients who have received an ICD for the primary and secondary prevention of Sudden Cardiac Death (SCD) [6,7].

The aim of the study was to assess the impact of frailty syndrome on the increase in the number of device-related concerns after the implantation of an implantable cardioverter defibrillator.

## 2. Material and Methods 

### 2.1. Study Design and Settings

This was an observational, prospective, and cross-sectional study, which was conducted in the Department of Electrocardiology and Heart Failure. The study involved 103 patients (85 men and 18 women, average age 71.56 ± 8.17 years). A cardioverter defibrillator had been implanted in all of the patients a minimum of six months earlier.

### 2.2. Study Participants and Selection

The study sample was a group of 103 consecutive patients with an implanted single- or dual-chamber cardioverter defibrillator, who had been hospitalized in the Department of Electrocardiology and Heart Failure to exchange their ICD. 

The patients that were included were over 60 years of age and had had an implantable single- or dual-chamber cardioverter defibrillator for more than six months. All of the patients agreed to participate in the study. 

Patients were excluded if cardiac resynchronization therapy using an implanted ICD (CRT-D) had previously been performed or when there were any indications of CRT. Patients with diagnosed cancer in the active phase or with a previously diagnosed mental illness or stroke or those who were unable to complete the survey questionnaires completely were also excluded.

The optimal pharmacological treatment of the existing cardiovascular diseases was confirmed in all of the patients. All of the patients included in the study underwent a standard physical examination, a 12-lead electrocardiogram, and echocardiography. 

### 2.3. Ethical Considerations

The local ethics committee approved the study protocol (KNW/0022/KB/36/18). The study protocol complied with the version of the Helsinki Convention that was current at the time that the study was designed. Participation in this study was anonymous and voluntary. All of the participants gave their consent at the beginning of the study, and they were also informed of its purpose and of the possibility to withdraw their participation at any stage. 

### 2.4. Research Instruments

In all of the patients that were included in the study, their concerns about an implantable cardioverter defibrillator were assessed using the ICD Concerns Questionnaire (ICDC), and the occurrence of frailty syndrome was assessed using the Tilburg Frailty Indicator scale (TFI).

The ICDC questionnaire consists of 20 questions on the 5-point Likert scale, where 0 means not at all to 5 very much. It is possible to obtain a general result for the number of fears in the range of 0–20 (number of concerns) and a result for the severity of the fears of 0–80 (increasing number of concerns). A higher score indicates more serious concerns. The number of concerns can be used individually or in combination to achieve the total number of concerns (maximum 100). The ICDC questionnaire is composed of two subscales: The first subscale—factor 1 assesses the perceived limitations, while the second subscale—factor 2 assesses any device-specific concerns. The psychometric properties of the original ICDC questionnaire were assessed and were good (Cronbach’s alpha 0.94 for the entire questionnaire) [8].

The Tilburg Frailty Indicator, which is based on the concept of the frailty model, is a tool that was developed by Gobbens et al. The scale consists of two parts: part A—concerns the determinants of frailty syndrome, while part B includes 15 questions about the presence of the major components of frailty. There are eight physical components, four emotional components, and three social components of frailty syndrome that are relevant subscales. The total TFI value can be within a range of 0 to 15 points, and frailty syndrome is recognized as being 5 points and above. The higher the score, the higher the level of FS [9,10].

## 3. Statistical Analyses

To check the normality of the data distribution, the Shapiro–Wilk test was used. Comparisons of the two groups were performed using the Student’s *t*-test when there was a normal distribution of a variable, while the distribution of the variables was analyzed using the Mann test. The chi2 test was also used for selected non-parametric data when applicable. The Wilcoxon test was used to compare the values for the same two groups (before and after). The Spearman correlation coefficient r was used to correlate the TFI with the number of concerns about an ICD. Logistic regression analysis was also used to predict the factors that were associated with concerns about an ICD. A receiver operating characteristic (ROC) curve analysis was used to evaluate the diagnostic performance of frailty syndrome. The area under the curve was calculated to reflect and compare the predictive value of the concerns about an ICD in each domain to discriminate the patients who were frail; Youden’s index was also applied [11]. The results were considered to be significant at *p*-values < 0.05. All of the presented analyses were performed using MedCalc (MedCalc Software, Ostend, Belgium).

## 4. Results

The average time after the implantation of a cardioverter defibrillator was about five years; 77.7% of the patients declared that they had more than two chronic diseases. The characteristics of the patients that were included in the study are presented in Table 1.

In the group of patients with an implanted cardioverter defibrillator, 75.73% had recognized FS (83.3% women, 74.1% men). The average TFI point value was 6.55 ± 2.67; in women it was 7.28 ± 2.58, and in men, it was 6.4 ± 2.67; the difference was not statistically significant *p* = 0.206. In the physical domain, the average value was 4.06 ± 1.79 out of 8 points, or 50.75% of the maximum score (the average value in women was 4.61 ± 1.58, in men, it was 3.94 ± 1.83; *p* = 0.152). In the psychological domain, the average value was 2.06 ± 1.1 out of 4 points, which was 51.5% of the maximum score (the average value in women was 2.11 ± 0.76, in men, it was 2.05 ± 1.16; *p* = 0.824). Finally, in the social domain, the average value was 0.44 ± 0.55 points for out of a maximum of 3, which was 14.7% of maximum score (the average value in women was 0.55 ± 0.61, in men, it was 0.41 ± 0.54; *p* = 0.319).

A lack of concerns related to life with an implantable cardioverter defibrillator was expressed by 30.34% of the patients. The detailed results are presented in Table 2. The total questionnaire score in the patients with an implanted cardioverter defibrillator was 34.06 ± 18.15, and was higher in patients with frailty syndrome compared to robust patients. The same relationship was observed in the number of concerns and the severity of the concerns. Analysis of factor 1 (perceived limitation factors) and factor 2 (device-specific concerns) showed that robust patients had fewer serious concerns about both factors. The data from the ICDC questionnaires are summarized in Table 3.

The correlations between the level of frailty and the concerns about an implantable cardioverter defibrillator showed that the higher the number of points that were obtained in the evaluation of frailty, the greater the seriousness of the concerns, the number of concerns and factors. Statistically significant correlations were found between the ICDC domains and frailty syndrome in the physical, psychological, and social domains. The greater the severity of frailty syndrome was in the domains, the greater the seriousness of the concerns and anxiety that were associated with life with an implanted ICD. The details are presented in Table 4. 

In the logistic regression analysis, the presence of frailty was strongly associated with the total questionnaire score (OR = 1.0265, *p* = 0.00426), the seriousness of the concerns (OR = 1.0417, *p* = 0.00451), and the device-specific concerns—factor 2 (OR = 1.0982, *p* = 0.00424). The detailed results are presented in Table 5. 

**Figure 1:**
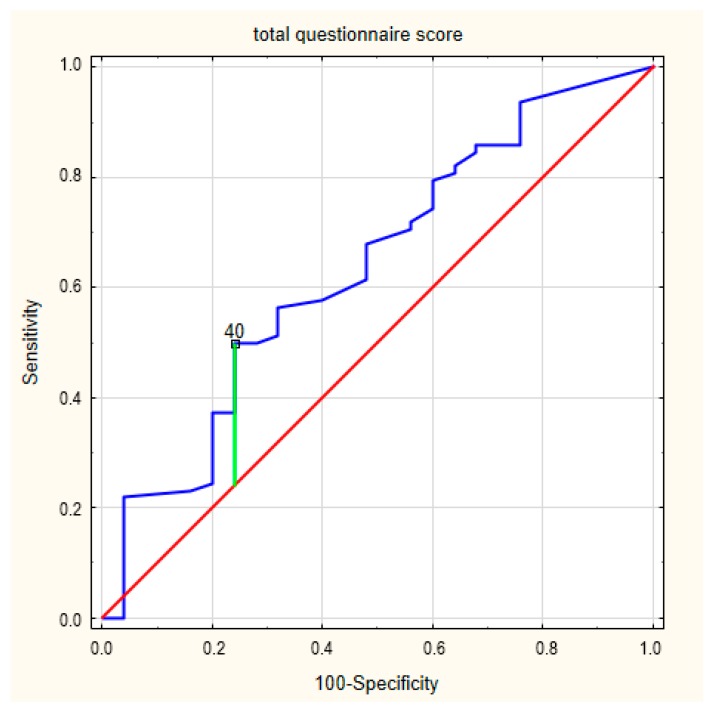
The receiver operating characteristic (ROC) curves for the concerns about an implantable cardioverter defibrillator (ICD) in frailty syndrome.

The ROC curves for the overall concerns about an ICD in frailty syndrome are presented in Figure 1. The area under the curve was 0.636 (95% CI = 0.535–0.728) The cut-off value for a designation of a concern about an ICD was 40 (*p* = 0.038). In this case, Youden’s index was 0.26. 

**Figure 2:**
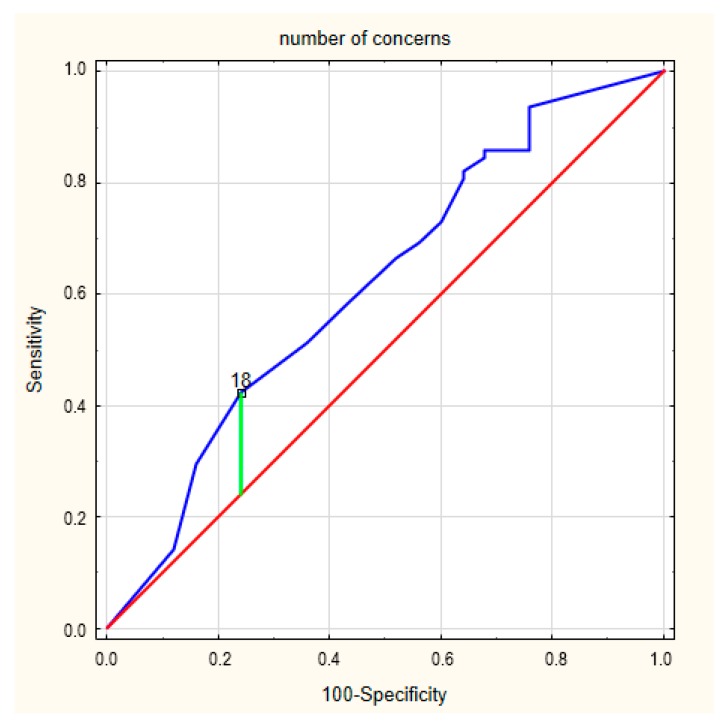
The ROC curves for the number of concerns in frailty syndrome.

**Figure 3:**
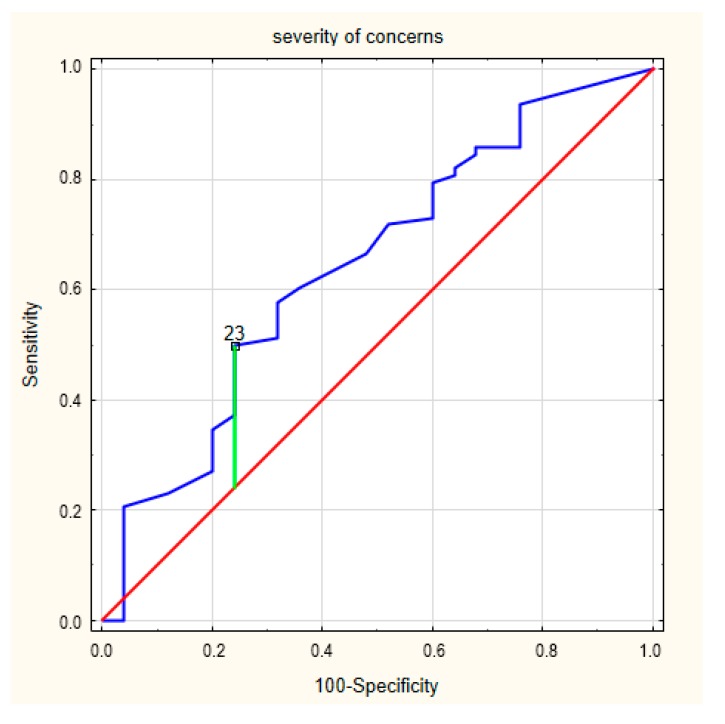
The ROC curves for the severity of the concerns in frailty syndrome.

Next, we analyzed the number and seriousness of concerns in ICD patients. The ROC curve for the number of concerns in patients in relation to the presence of frailty syndrome is presented in Figure 2. The area under the curve was 0.617 (95% CI = 0.516–0.711). The cut-off value for a designation of a concern about an ICD was 18 (*p* = 0.082), and Youden’s index was 0.18. Meanwhile, the ROC curve for the seriousness of the concerns in relation to the presence frailty is presented in Figure 3. The area under the curve was 0.644 (95% CI = 0.544–0.736). The cut-off value for the designation of a concern about an ICD was 23 (*p* = 0.027), and Youden’s index was 0.26. 

**Figure 4:**
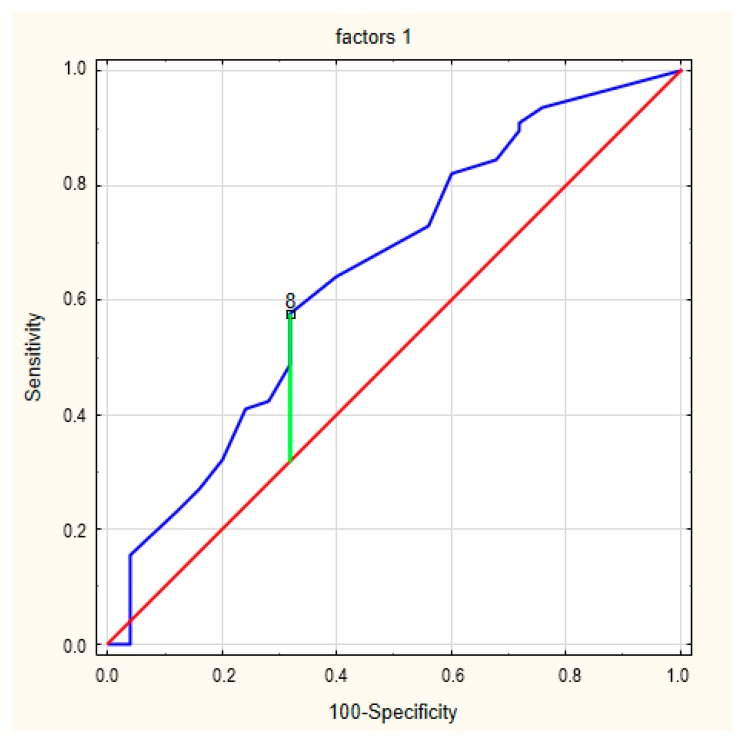
The ROC curves for factor 1 in frailty syndrome.

**Figure 5:**
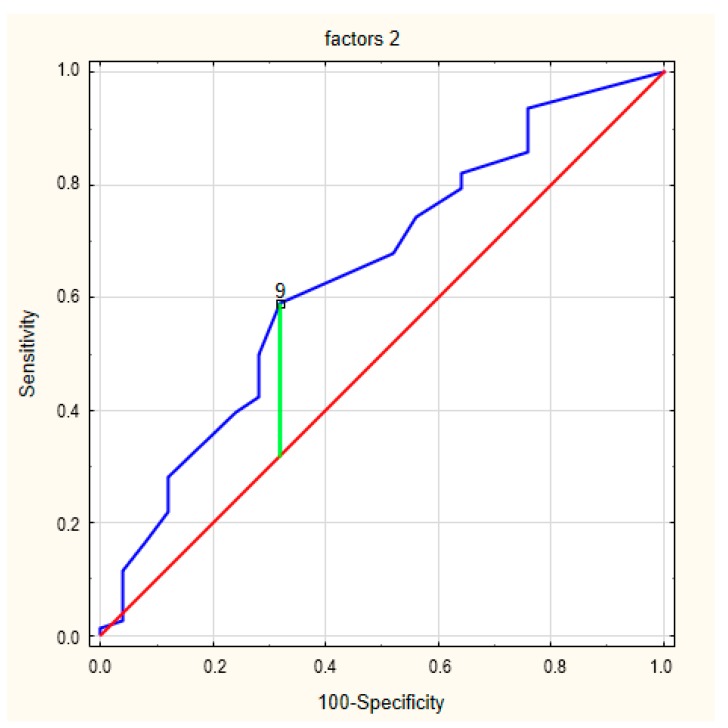
The ROC curves for factor 2 in frailty syndrome.

The final analysis concerned the perceived limitations (factor 1) and device-specific concerns (factor 2). The ROC curve for factor 1 in relation to frailty syndrome is presented in Figure 4. The area under the curve was 0.647 (95% CI = 0.547–0.739). The cut-off value for the designation of a concern about an ICD was 8 (*p* = 0.026), and Youden’s index was 0.26. The ROC curve for factor 2 in frailty syndrome is presented in Figure 5. The area under the curve was 0.643 (95% CI = 0.543–0.735). The cut-off value for the designation of a concern about an ICD was 9 (*p* = 0.0272), and Youden’s index was similar, 0.27. 

## 5. Discussion

The results of the current study indicate that patient concerns about life after ICD implantation are prevalent. Patients’ concerns about an ICD were a determinant of frailty syndrome. Frailty is a serious problem in older people [12]. Our result suggests that having a large number of ICD-related concerns has a greater impact on physical, psychological, and social frailty. This observation is important because some other studies have shown that performing a holistic geriatric assessment and diagnosis of frailty syndrome can result in adequate intervention and reduce the severity of the concerns about an implanted ICD. The results of our study have practical clinical implications for the elderly population, especially in addressing the problem of the fear of future shocks. An assessment of the problems that are associated with an implantable ICD can be performed in clinical practice using the short instruments that were used in this study. Because concerns about an implanted ICD are not constant and may change over time, it is important to evaluate these concerns regularly [13,14]. In the study of Kramer et al., the authors diagnosed frailty syndrome in 12.8% of the patients with an implanted ICD and prefrail in 47.5% of the patients. Only 39.7% of the patients in the cited research were robust. In our study, we identified frailty syndrome in 75.73% of the patients. The difference can be explained by the use of a different questionnaire that does not offer a diagnosis of prefrail and the older age of the patients that were included in the study [15].

Limiting or avoiding previously enjoyed activities, hobbies, etc., and limitations in their physical functioning may be some of the reasons why a significant proportion of the ICD patients had anxiety and concerns about the implanted device. In our study, we showed that the severity of frailty syndrome in the physical, psychological, and social domains resulted in an increase in ICD-related concerns, but only in the population that was more than 60 years old. Patients without diagnosed frailty syndrome had fewer concerns in each of the analyzed domains [16].

Although frailty syndrome is a common syndrome among elderly patients with cardiovascular disease, including arrhythmias, frailty syndrome has not yet been shown to increase the concerns that are associated with an implanted ICD. Studies have shown that patients with diagnosed frailty syndrome show symptoms of anxiety and depression more often, and this can increase the incidence of ICD-related concerns. A diagnosis of frailty syndrome could contribute to an earlier diagnosis of the disorders that are related to mood swings, concern, or anxiety [17,18].

In older adults with coexisting illnesses, ICD therapy may affect everyday life because of potential ICD-related complications, an increased number of hospitalizations, inappropriate shocks, etc. [19]. In this context, Green et al. stressed the important role of frailty syndrome screening among older adults who are being considered for the implantation of primary prevention ICDs [20]. Similar to our paper, Green also concluded that frailty screening could improve the appropriate use of ICDs as well as patient outcomes.

## 6. Conclusions

To summarize, it was found that frailty syndrome occurred in about 76% of the patients after ICD implantation. This had a negative effect on the concerns in all of the domains (total questionnaire score, number of concerns, seriousness of concerns, factor 1—perceived limitations and factor 2—device-specific concerns) among those patients. Frailty syndrome was strongly associated with concerns about an implantable cardioverter defibrillator.

### Study limitations

The study had a few potential limitations. One of those limitations was the fact that our sample was relatively small and was recruited from only one center. In addition, an intensification of concerns in connection with an implantation ICD may be affected by a subjective assessment of the quality of life and the clinical condition of a patient. Another limitation of this study could have been the use of only one tool to identify frailty syndrome. There is a lack of guidelines for selecting a specific tool for assessing a patient’s FS after ICD implantation in clinical practice. In addition, the survey covered a larger number of men, although consecutive patients were enrolled in the study. It seems appropriate to continue the study on a larger group of patients, including women. Despite these restrictions, this is one of the first researches on the relationship between frailty syndrome and a patient’s concerns related to functioning with an implanted ICD.

## Figures and Tables

**Table 1:** Characteristics of the patients that were included in the study.

Parameter	All	Frail	Robust	*p*
Age	71.56 ± 8.17	71.67 ± 8.40	71.24 ± 7.54	0.562
Weight	80.89 ± 17.61	79.01 ± 18.13	86.28 ± 15.19	0.386
BMI	28.80 ± 4.72	28.04 ± 4.83	30.25 ± 4.49	0.748
Place of residence				
City area	81.55%	79.49%	88%	0.339
Rural area	18.45%	20.51%	12%	
Education				
Non or primary	37.87%	61.54%	56%	0.693
Secondary	61.16%	1.28%	4%	
Vocational or higher	0.97%	37.18%	40%	
Marital Status				
Married/living with partner	89.32%	88.46%	92%	0.683
Unmarried	7.77%	8.97%	4%	
Widow/widower	2.91%	2.56%	4%	
Professional status				
Working	21.36%	19.23%	28%	0.568
Retired	71.84%	73.08%	68%	
Pensioner	6.80%	7.69%	4%	
Smoking				
Smoker	29.13%	44%	24.36%	0.059

**Table 2:** Characteristics of the severity of the concerns about the implanted cardioverter defibrillator.

Number	Question I Am Worried About:	Not at all n (%)	A little Bit n (%)	Somewhat n (%)	Quite a Lot n (%)	Very Much So n (%)
1.	My ICD firing	21 (20.39%)	52 (50.48%)	27 (26.21%)	3 (2.91%)	0 (0%)
2.	My ICD not working when I need it to	26 (25.24%)	55 (53.40%)	18 (17.47%)	4 (3.88%)	0 (0%)
3.	What I should do if my ICD fires	29 (28.16%)	46 (44.66%)	26 (25.24%)	2 (1.94%)	0 (0%)
4.	Doing exercise in case it causes my ICD to fire	29 (28.16%)	42 (40.78%)	29 (28.16%)	3 (2.91%)	0 (0%)
5.	Doing activities/hobbies that may cause my ICD to fire	28 (27.18%)	45 (43.69%)	26 (25.24%)	4 (3.88%)	0 (0%)
6.	My heart condition getting worse if the ICD fires	27 (26.21%)	40 (38.83%)	32 (31.07%)	4 (3.88%)	0 (0%)
7.	The amount of time I spend thinking about my heart condition and having an ICD	33 (32.04%)	43 (41.75%)	26 (25.24%)	1 (0.97%)	0 (0%)
8.	The amount of time I spend thinking about my ICD firing	32 (31.07%)	40 (38.83%)	28 (27.18%)	3 (2.91%)	0 (0%)
9.	The ICD battery running out	27 (26.21%)	41 (39.81%)	25 (24.27%)	10 (0.97%)	0 (0%)
10.	Working too hard/overdoing things and causing my ICD to fire	30 (29.13%)	41 (39.81%)	29 (28.16%)	3 (2.91%)	0 (0%)
11.	Making love in case my ICD fires	34 (33.01%)	44 (42.72%)	21 (20.39%)	4 (3.88%)	0 (0%)
12.	Having no warning that my ICD will fire	26 (25.24%)	36 (34.95%)	36 (34.95%)	5 (4.85%)	0 (0%)
13.	The symptoms/pain associated with my ICD firing	24 (23.30%)	45 (43.69%)	31 (30.09%)	3 (2.92%)	0 (0%)
14.	Being a burden on my partner/family	48 (46.60%)	35 (33.98%)	16 (15.53%)	4 (3.88%)	0 (0%)
15.	Not being able to prevent my ICD from firing	27 (26.21%)	45 (43.69%)	26 (25.24%)	4 (3.88%)	1 (0.97%)
16.	The future now that I have an ICD	22 (21.36%)	48 (46.60%)	30 (29.13%)	3 (2.91%)	0 (0%)
17.	Problems occurring with my ICD, e.g., battery failure	31 (30.09%)	42 (40.77%)	25 (24.27%)	5 (4.85%)	0 (0%)
18.	Getting too stressed in case my ICD fires	29 (28.16%)	54 (52.43%)	18 (17.47%)	2 (1.94%)	0 (0%)
19.	Not being able to work/take part in activities and hobbies because I have an ICD	46 (44.66%)	31 (30.09%)	22 (21.36%)	4 (3.88%)	0 (0%)
20.	Exercising too hard and causing my ICD to fire	56 (54.37%)	35 (33.98%)	11 (10.68%)	1 (0.97%)	0 (0%)

**Table 3:** Intensification of the concerns depending on the occurrence of frailty syndrome.

Parameter	Frail	Robust	All	*p*
Total questionnaire score	36.14 ± 17.08	27.56 ± 20.13	34.06 ± 18.15	0.039035
Number of concerns	14.55 ± 5.85	11.68 ± 7.49	13.85 ± 6.37	0.049406
Severity of concerns	21.59 ± 11.65	15.88 ± 13.23	20.20 ± 12.23	0.041722
Factor 1—perceived limitations	8.93 ± 5.12	6.48 ± 5.92	8.34 ± 5.40	0.047413
Factor 2—device-specific concerns	9.63 ± 5.23	7.08 ± 5.52	9.01 ± 5.39	0.038913

**Table 4:** Correlations between the level of frailty and the concerns about the implantable cardioverter defibrillator.

Parameter	Frailty Syndrome Global	Physical Domain	Psychological Domain	Social Domain
Total questionnaire score	r = 0.5090	r = 0.4804	r = 0.3340	r = 0.2274
	*p* = 0.000	*p* = 0.000	*p* = 0.001	*p* = 0.021
Number of concerns	r = 0.4744	r = 0.4236	r = 0.3506	r = 0.2125
	*p* = 0.000	*p* = 0.000	*p* = 0.000	*p* = 0.031
Severity of concerns	r = 0.5079	r = 0.4920	r = 0.3128	r = 0.2267
	*p* = 0.000	*p* = 0.000	*p* = 0.001	*p* = 0.021
Factor 1—perceived limitations	r = 0.4972	r = 0.4885	r = 0.2801	r = 0.2511
	*p* = 0.000	*p* = 0.000	*p* = 0.004	*p* = 0.011
Factor 2—device-specific concerns	r = 0.4917	r = 0.4647	r = 0.3223	r = 0.2186
	*p* = 0.000	*p* = 0.000	*p* = 0.001	*p* = 0.027

**Table 5:** Logistic regression analysis of frailty and the concerns after implantable cardioverter defibrillator (ICD) implantation.

	Rating	Standard Error	Wald x^2^	OR	95% CI	*p*
Total questionnaire score	0.026153	0.012898	4.1117	1.0265	1.0009–1.0528	0.0426
Number of concerns	0.065835	0.034166	3.7126	1.0680	0.9989–1.1420	0.0540
Severity of concerns	0.040839	0.020379	4.0161	1.0417	1.0009–1.0841	0.0451
Factor 1—perceived limitations	0.091981	0.047114	3.8115	1.0963	0.9996–1.2024	0.0509
Factor 2—device-specific concerns	0.093687	0.046153	4.1205	1.0982	1.0032–1.2022	0.0424
